# Periprocedural Use of Heparin and Other Factors Contributing to the Patency of the Radial Artery Following Diagnostic Coronary Angiography

**DOI:** 10.3390/jcm14051401

**Published:** 2025-02-20

**Authors:** Tomasz Bochenek, Adam Pytlewski, Michał Lelek, Bartosz Gruchlik, Jan Szczogiel, Marek Grabka, Andrzej Jaklik, Maciej Podolski, Katarzyna Mizia-Stec

**Affiliations:** 1 First Department of Cardiology, Medical University of Silesia, 40-635 Katowice, Poland; michlel@wp.pl (M.L.); bartosz1301@gmail.com (B.G.); janszczo@wp.pl (J.S.); marekgrabka@interia.pl (M.G.); ajaklik@onet.pl (A.J.); maciekmcee@onet.eu (M.P.); kmiziastec@gmail.com (K.M.-S.); 2 European Reference Network for Rare, Low Prevalence, or Complex Diseases of the Heart (ERN GUARD Heart), 81377 Munich, Germany; 3 Bielanski Hospital, 01-809 Warsaw, Poland; a.pytlewski@gmail.com

**Keywords:** radial artery occlusion (RAO), unfractionated heparin, diagnostic angiography, vascular sheath, catheters, Doppler ultrasound examination, atherosclerotic changes, compression time, spasmolytics, AI

## Abstract

**Background:** Diagnostic angiography of coronary arteries is one of the most common procedures in invasive cardiology and is mainly performed via the radial artery. Rapid improvements in the quality of the equipment and operator’s experience have changed the landscape of this procedure. **Methods:** In this study, 284 patients were analyzed to determine whether heparin administration should be a necessity for all patients to prevent radial artery occlusion (RAO). Moreover, the possible influence of 51 other factors on RAO was analyzed. **Results:** This study revealed that heparin administration does not have a significant impact on RAO incidence (*p* = 0.131). However, it was found that a longer fluoroscopy time (*p* < 0.001) and smaller (5F) vascular sheath (*p* = 0.001) might serve as protective factors against RAO. On the other hand, a greater number of catheters (*p* < 0.001), greater compression time (*p* = 0.001), wider (6F) vascular sheath (*p* = 0.002), spasm occurrence (*p* = 0.001), spasmolytic administration (*p* < 0.001) and atherosclerotic changes in the radial artery (*p* = 0.005) were risk factors for RAO. **Conclusions:** This study demonstrates the need for a more personalized approach to the patient when analyzing the individual risk of RAO. In our opinion, it is possible to omit heparin in cases of patients with an initial low risk of RAO or possible adverse drug reactions during diagnostic angiography.

## 1. Introduction

### 1.1. Study Purpose and Background

The diagnostic angiography of coronary arteries is one of the most common procedures in invasive cardiology. The use of computed tomography has, however, lowered the number of these procedures in recent years. Most invasive procedures are performed from the right or left radial artery (RA). In recent years, the deliverability, flexibility and size of the equipment used in coronary angiography have improved. Operators have significantly increased in experience. All those factors undoubtedly reduce endothelial injury to radial artery, reduce risk of spasm and reduce the possibility of consecutive arterial occlusion. Moreover, the procedures are shorter and patient comfort is much better. In light of the fast expansion of noninvasive angiography, the risk of invasive procedures has to be minimized. Operators are already gradually abandoning the spasmolytic agents that have been used in practice for years, which was the subject of our previous research. Heparin is still usually used routinely for diagnostic angiography. However, some operators have stopped using this drug in sole uncomplicated diagnostic coronary angiography. With all its potential side effects, hypersensitivity in certain groups of patients and contraindications to heparin, the purpose of this study was to explore whether this drug should still be mandatory for all relevant patients qualified to receive diagnostic angiography.

### 1.2. Aim

The main aim of this study was to assess whether heparin reduces the risk of radial artery occlusion (RAO) during diagnostic angiography in patients treated with antiplatelet drugs. The secondary aim was to analyze other possible factors influencing the patency of the artery during the procedure.

## 2. Materials and Methods

### 2.1. Study Group

**Figure 1:**
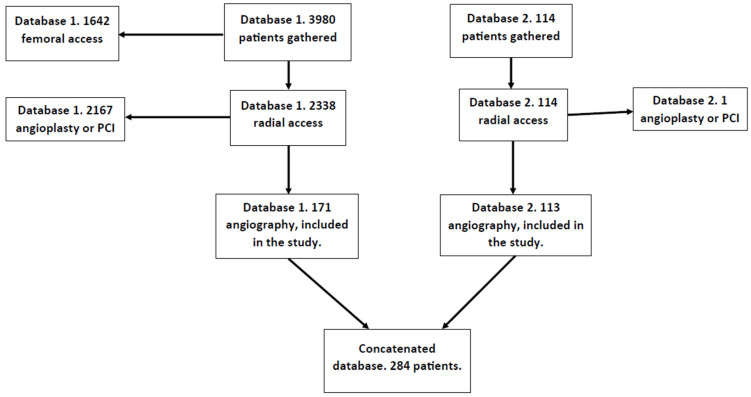
Database creation flowchart.

This was a single-center study, and the data were analyzed retrospectively. Two databases were concatenated and analyzed together. The first database was the subject of our previous study. From this database, 171 patients were recruited. The study for which the database was originally created did not need separate bioethical commission approval according to the regulator’s document KNW/0022/KB/69/18. The second database included 113 patients, the majority of whom (92%) did not receive heparin during the procedure upon the operator’s decision. To gather the data for this database, bioethical commission approval with the number PCN/CBN/0052/KB1/90/22 (20 September 2022) was obtained. Both databases were assembled at the 1st Department of Cardiology of the Upper Silesian Medical Center and covered the period from October 2016 to January 2018 and the period from September 2022 to October 2023, respectively. This study was performed on adults treated with a single antiplatelet agent. Exclusion criteria were an angioplasty with or without stent implantation and diagnostic studies requiring heparin (intravascular ultrasound; optical coherence tomography; fractional flow reserve). These patients were given heparin mandatorily and were not included in the study. The final number of patients included in the analysis was 284. The process of obtaining the data for the database is presented in the flowchart in Figure 1.

### 2.2. Procedural Steps for the Radial Access

Before the procedure, patients had their pulse palpated on radial artery.

Treatments were performed through the radial artery without premedication prior to the procedure. To numb the puncture site of the radial artery, the patient was given 2 mL of lidocaine through subcutaneous injection. Radial artery access was achieved with the use of open bore needles, 0.025″ hydrophilic short guidewires and 6 Fr or 5 Fr Balton vascular access sheaths. In the first group of analyzed patients, 5000 IU of unfractionated heparin was injected as a bolus immediately after insertion of the sheath. The second group of patients were those selected patients who did not receive heparin at all based on the decision of the operator.

In all the procedures, operators used 0.035″ guides for the insertion of catheters and standard 5F or 6F catheters. The vascular sheath was removed immediately after the procedure, and to obtain full homeostasis of the access site, standard tourniquets were used, which were inflated with air and removed after the required amount of time.

### 2.3. Postprocedural Assessment of Patency

On the day after the procedure, patients underwent an ultrasound assessment. The radial and brachial arteries’ diameters were measured. Radial artery imaging was performed after the procedure in cross-sectional views by placing the ultrasound 2 cm proximally to the styloid process of the radius perpendicular to the arterial wall, over the puncture site. Patency was determined using the presence of a biphasic or triphasic signal in the Doppler ultrasound examination. The lack of these signals indicated occlusion of the RA.

### 2.4. Statistical Analysis

Statistical analysis was performed using the R programming language (version 4.3.2) with RStudio Integrated Development Environment 2023.12.1 Build 402. The statistical significance level was set to 0.05. Data analysis included computing mean and standard deviation for numerical variables and percentage values for categorical variables. Univariate logistic regression analysis was performed, with the patency of the radial artery being the dependent variable. Variables with statistically significant coefficients had their odds ratios with 95% confidence intervals computed.

## 3. Results

### 3.1. Clinical Characteristics

The data of 286 patients were analyzed. Mean age was 67.5 years; 54% of the patients were males. Most of the patients presented with ischemic heart disease (68%), arterial hypertension (82%) and hyperlipidemia (65%). A large portion of patients suffered from diabetes (31%), had a history of myocardial infarction (20%) and had previous percutaneous coronary interventions (21%). In total, 87% of the patients (n = 249) had a patent radial artery, whereas 13% (n = 37) suffered from occlusion of the radial artery after percutaneous intervention. A total of 52 characteristics of the patients were analyzed. Table 1 presents a complete list of the numerical independent variables. Table 2 presents a complete list of the categorical independent variables.

### 3.2. Group Differences

**Figure 2:**
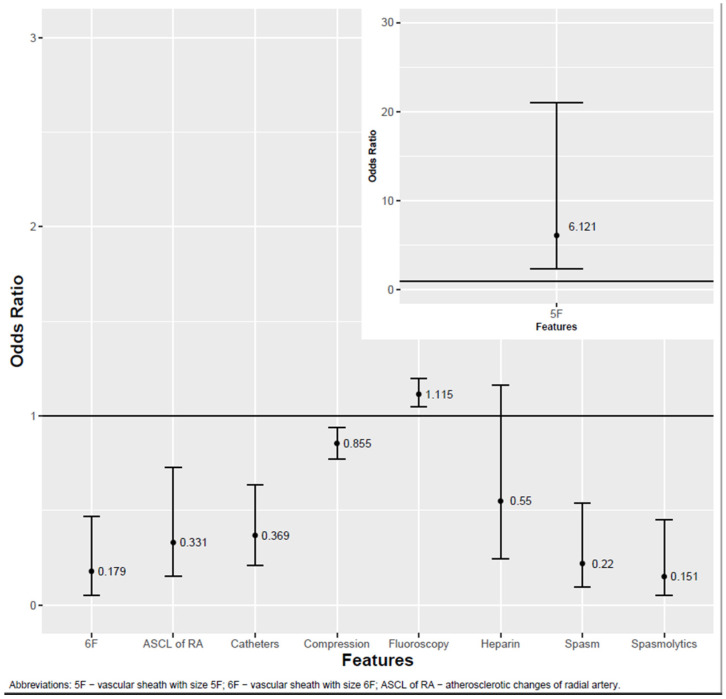
Factors influencing RA patency.

Of the 52 variables, 8 had a significant association with the occlusion. Of these eight variables, two had negative and six had positive associations. A longer fluoroscopy time (*p* < 0.001) and 5F vascular sheath (*p* = 0.001) were associated with lower chances of RAO. A greater number of catheters used (*p* < 0.001), greater compression time (*p* = 0.001), 6F vascular sheath (*p* = 0.002), spasm occurrence (*p* = 0.001), spasmolytic administration (*p* < 0.001) and atherosclerotic changes in the radial artery (*p* = 0.005) were associated with a greater risk of occlusion. Heparin administration (*p* = 0.131) was not associated with occlusion. The study did not find any significant association between RAO and patients’ chronic diseases or medical history. Anatomical features such as the origin of the brachial artery, RA diameter and pathological intima–media complex had no associations with the incidence of RAO. The crucial results are presented in Figure 2, whereas the results for all variables are presented in Table 3.

## 4. Discussion

A single-center retrospective study was conducted to assess which factors could potentially influence the occlusion of the radial artery after diagnostic percutaneous coronary assessment. The data of 286 patients were analyzed. It was found that a longer fluoroscopy time and smaller (5F) vascular sheath were associated with lower chances of RAO. In contrast, a greater number of catheters used, longer compression time, larger (6F) vascular sheath, spasm occurrence, spasmolytic administration and atherosclerotic changes in the radial artery were associated with an increased risk of occlusion.

Heparin administration was found to have no statistically significant association with the risk of RAO. However, two meta-analyses and one review showed contrary results [1,2,3]. Rashid et al. [1] found that the risk of RAO is lower when the dose of heparin is higher (5000 vs. 2000–3000 IU). Moreover, the study reported that a higher dose of heparin did not result in a greater risk of bleeding. It also found a lower incidence of RAO in PCI than in diagnostic coronarography. Dahal et al.’s [2] meta-analysis compared a standard dose (5000 IU) of heparin administration with a lower dose (<2500 IU). The results showed the same finding: a higher dose of heparin resulted in a lower risk of RAO. However, a greater dose of heparin was associated with a higher risk of hematoma. Avdikos et al. [3] mentioned that the influence of anticoagulant agents as a protective factor against RAO has been investigated. The study cited the findings of Spaulding et al. [4], who diagnosed RAO in 71% of patients who did not receive heparin, 24% of patients who received 2000–3000 IU of heparin and 4.3% of patients who received 5000 IU of heparin. Bernat et al. [5] compared the risk of RAO in patients who were administered a lower (2000 IU) or higher (5000 IU) dose of unfractionated heparin. The incidence of final RAO remained significantly lower after a higher dose of heparin. It is hard to explain why our study showed different results: potentially, the lower number of participants and retrospective nature of the study design made it more prone to bias. However, we think that modern tools in cardiology and the fact that the procedures in our study were performed by qualified operators also influenced our results. Experienced invasive cardiologists not only usually have more success in radial artery puncture but also limit the device movements significantly, which may be crucial to the level of mechanical trauma caused to the artery. Our aim was not to question the use of heparin in angiography in all patients; we just wanted to establish whether it would be safe to perform the procedure without heparin in a certain group of patients who would potentially be prone to adverse events or could not take the drug at all, without increasing the likelihood of radial artery occlusion. Our findings could potentially be further evaluated in the light of new tools/AI and increasing operator experience in performing coronary angiography via the radial artery. In the future, heparin will perhaps only be used if the duration of the procedure is prolonged or more catheters are used.

We found that the size of the vascular sheath was associated with RAO incidence. The use of a vascular sheath with a smaller diameter was associated with a lower risk of RAO, whereas a greater diameter was associated with a greater risk of RAO. This phenomenon has also been mentioned in other studies [6,7,8,9]. Goswami et al., in their review [6], pointed out that an important risk factor of RAO is a ratio of the radial artery’s inner diameter to the sheath’s outer diameter of less than 1, and RAO prevention should include the use of slender sheaths. Dwivedi et al. [7] noted that a larger vascular sheath size is an independent risk factor of RAO. Buturak et al. [8] reported that a greater sheath-to-artery diameter correlates with a greater risk of RAO; however, the use of 5F or 6F sheaths without information about the radial artery diameter has no direct impact on RAO. Conversely, Rashid et al., in their meta-analysis [1], reported that even though the size of the catheter could possibly correlate with the risk of RAO, the final results did not show significant benefits from using more slender or larger catheter sheaths. Rashid et al. also pointed out that studies researching the influence of vascular sheaths were often poorly randomized and thus sensitive to selection bias. Another important observation was that these studies often did not include the diameter of the radial artery. This systematic review reports the need for large, well-randomized studies that include the measurement of the radial artery diameter and sheath-to-artery ratio.

A longer compression time was positively associated with the incidence of RAO. Rashid et al.’s meta-analysis [1] analyzed the studies of Chou et al. [10] and Politi et al. [11] with the same finding. Both studies’ results were significant yet underpowered, with the number of patients being 100 for Chou et al. [10] and 120 for Politi et al. [11]. Dharma et al.’s study [12], which featured 1706 participants, showed that prolonged (>4 h) compression time is a strong predictor of RAO.

Another interesting finding was that a longer fluoroscopy time was negatively associated with RAO. Other studies reporting this phenomenon have not been found. Buturak et al. [8] and Uhlemann et al. [9] mentioned fluoroscopy time as a non-statistically important factor. It is hard to discern why this particular variable proved to be important in our study. A possible explanation is that a greater fluoroscopy time resulted in better visual control, thus allowing for more precise movements and catheter manipulation during the procedure. For example, when passing wires or catheters through the radial artery, a more careful operator could reduce the amount of damage to the endothelium. Procedural expertise could be an additional protective factor, yet it was not measured in this study.

Atherosclerotic changes in the RA were positively associated with RAO. This factor has been analyzed in two studies [9,13]: Uhlemann et al. [9] and Dangoisse et al. [13] found similar results.

Spasm and the administration of spasmolytic drugs were positively associated with RAO. We performed an additional analysis, where these factors were strongly correlated (Crammer’s V = 0.66 and *p* < 0.001): half of the patients with spasm received spasmolytics, and only one participant without spasm received spasmolytics. Thus, we believe that spasm occurrence could increase the risk of RAO, yet the influence of spasm on occlusion has also been reported in other studies [14,15].

A greater catheter count was positively associated with RAO. A similar discovery has been reported by Sadaka et al. [16] This is probably because subsequent catheter introduction can increase the risk of damaging the endothelium, which can later lead to occlusion.

The study found that patients’ age, sex, chronic diseases as well as medical history had no associations with RAO incidence. Rashid et al.’s [1] meta-analysis mentions several studies which reported age, sex, body weight, RA diameter and several other factors as potential predictors of RAO. However, these findings were not consistent across all the studies, and a meta-analysis did not find a consistent direction for these variables’ effect.

Important limitations of this study include its retrospective character and the limited number of participants. Hahalis et al. [17] conducted a similar study with randomization and a prospective design, comparing standard and higher heparin doses when performing diagnostic coronary angiography. The study showed contrary results to ours: a higher dose of heparin reduces the incidence of RAO. However, we found that Hahalis et al. [17] used only a percentage of patients on prior antiplatelet treatment, contrary to this study, where one of our inclusion criteria was for patients to have at least one antiplatelet agent chronically prior to the procedure.

## 5. Conclusions

This study explored potentially important factors associated with RAO. We found that a smaller vascular sheath size and longer fluoroscopy time could favor the patency of the RA. In contrast, a greater number of catheters used, longer compression time, spasm occurrence, spasmolytic administration and atherosclerotic changes in the RA were associated with a higher risk of RAO. Heparin administration had no association with the occlusion risk in our group of patients. These results provide suggestions for what we can change to reduce the risk of RAO. Some of the results, such as the influence of fluoroscopy time or the lack of influence of heparin, are new findings that could be further evaluated. In concordance with the results, the authors of the study claim that, at present, in certain groups of patients with sole uncomplicated radial artery angiography, heparin could be omitted safely without increasing the risk of radial artery occlusion. This might be the subject of future research.

## Figures and Tables

**Table 1:** Characteristics of the participants (continuous variables).

Feature	All Patients (n = 286)	Patients with Occluded Radial Artery (n = 37)	Patients with Patent Radial Artery (n = 249)	*p*-Value
Age (years)	67.50 ± 9.98	64.89 ± 11.47	67.87 ± 9.72	0.164
Height (cm)	166.39 ± 11.12	167.14 ± 10.11	166.29 ± 11.27	0.812
Weight (kg)	80.55 ± 15.47	82.92 ± 15.36	80.22 ± 15.49	0.454
Creatinine (mg/dL)	0.96 ± 0.38	0.98 ± 0.24	0.96 ± 0.40	0.34
eGFR (ml/min/1.73 m^2^)	74.37 ± 15.67	73.11 ± 14.96	74.55 ± 15.79	0.43
EF (%)	51.53 ± 10.92	50.23 ± 11.63	51.71 ± 10.82	0.645
Catheters count (n)	1.60 ± 0.63	1.97 ± 0.79	1.55 ± 0.59	0.001
Coronarography time (min)	30.29 ± 11.47	28.15 ± 12.63	30.60 ± 11.29	0.223
Fluoroscopy time (min)	9.76 ± 7.56	5.71 ± 5.90	10.35 ± 7.61	<0.001
Fluoroscopy dose (cGy∙cm^2^)	204.14 ± 210.32	185.53 ± 138.45	206.80 ± 218.75	0.724
Iodinated contrast administered (mL)	63.60 ± 41.30	59.71 ± 22.89	64.14 ± 43.26	0.765
Pain level (1–10 scale)	3.83 ± 2.33	4.52 ± 2.64	3.75 ± 2.28	0.143
Compression time (min)	5.88 ± 3.08	7.93 ± 3.73	5.62 ± 2.90	<0.001
Brachial artery diameter (mm)	5.05 ± 0.74	5.05 ± 0.86	5.05 ± 0.72	0.598
Maximal diameter of radial artery (mm)	3.18 ± 0.51	3.22 ± 0.80	3.18 ± 0.47	0.278
Minimal diameter of radial artery (mm)	2.74 ± 0.47	2.75 ± 0.83	2.73 ± 0.41	0.891

Data are presented as mean ± SD, number of patients or percentages. Abbreviations: SD—standard deviation; eGFR—estimated glomerular filtration rate; EF—ejection fraction.

**Table 2:** Characteristics of the participants (categorical variables).

Feature	All Patients (n = 286)	Patients with Occluded Radial Artery (n = 37)	Patients with Patent Radial Artery (n = 249)	*p*-Value
Sex (% of males)	54.26	54.29	54.25	1
Recognized IHD (%)	68.44	68.57	68.42	1
Diabetes (%)	30.5	31.43	30.36	1
Hypertension (%)	82.27	74.29	83.4	0.234
Hyperlipidemia (%)	65.25	51.43	67.21	0.087
Smoking (%)	23.05	17.14	23.89	0.52
History of CVA (%)	7.45	0	8.5	0.087
History of MI (%)	19.86	22.86	19.43	0.652
PCI performed previously (%)	18.09	22.86	17.41	0.481
History of PCI (%)	21.28	25.71	20.65	0.51
History of CABG (%)	4.61	0	5.26	0.381
Diagnosis on admission: arteriosclerosis (%)	29.08	20	30.36	0.238
Diagnosis on admission: unstable angina (%)	16.31	5.71	17.81	0.086
Diagnosis on admission: NSTEMI (%)	5.67	0	6.48	0.234
Diagnosis on admission: HD (%)	13.48	11.43	13.77	1
Diagnosis on admission: other (%)	16.67	14.29	17	0.812
Right radial artery (%)	94.98	91.18	95.51	0.39
Heparin administered (%)	59.57	71.43	57.89	0.144
Coronarography conducted (%)	98.58	100	98.38	1
IVUS examination conducted (%)	3.91	2.86	4.07	1
FFR measurement conducted (%)	4.96	5.71	4.86	0.688
Vascular sheath 5F (%)	40.07	11.43	44.13	<0.001
Vascular sheath 6F (%)	61.92	88.57	58.13	<0.001
Catheter 6F (%)	36.88	31.43	37.65	0.576
TIG catheter (%)	11.35	14.29	10.93	0.569
Spasm occurrence (%)	10.64	28.57	8.1	0.001
Spasmolytic administered (%)	5.67	20	3.64	0.001
Relanium administered (%)	2.49	2.86	2.44	1
Isoptin administered (%)	0.36	0	0.41	1
NTG administered (%)	2.49	5.71	2.03	0.212
Conversion to transfemoral access (%)	2.14	2.86	2.03	0.553
Pathological IMT (%)	71.37	71.43	71.37	1
High origin of the radial artery (%)	6.93	6.9	6.94	1
Atherosclerotic changes in radial artery (%)	19.57	38.24	17	0.009
Presence of hematoma (%)	5.69	0	6.48	0.232
False aneurysm (%)	1.07	0	1.21	1

Data are presented as percentages. Abbreviations: IHD—ischemic heart disease; CVA—cerebral vascular accident; PCI—percutaneous coronary intervention; CABG—coronary artery bypass graft surgery; MI—myocardial infarction; HD—heart defect; IVUS—intravascular ultrasound; FFR—fractional flow reserve; TIG—tiger; NTG—nitroglicerin; IMT—intima-media complex thickness.

**Table 3:** Results of the analysis.

Feature	OR	*p*-Value
Continuous features		
Age (years)	1.029 (0.994–1.066)	0.099
Height (cm)	0.993 (0.956–1.025)	0.684
Weight (kg)	0.989 (0.966–1.013)	0.355
Creatinine (mg/dL)	0.888 (0.422–2.621)	0.778
eGFR (mL/min/1.73 m^2^)	1.006 (0.983–1.027)	0.613
EF (%)	1.012 (0.98–1.042)	0.452
Catheters count (n)	0.369 (0.208–0.634)	<0.001
Coronarography time (min)	1.023 (0.989–1.064)	0.232
Fluoroscopy time (min)	1.115 (1.047–1.2)	0.002
Fluoroscopy dose (cGy∙cm^2^)	1.001 (0.999–1.003)	0.576
Iodinated contrast administered (mL)	1.004 (0.995–1.019)	0.559
Pain level (1–10 scale)	0.875 (0.748–1.027)	0.096
Compression time (min)	0.855 (0.774–0.937)	0.001
Brachial artery diameter (mm)	0.998 (0.568–1.673)	0.993
Maximal diameter of radial artery (mm)	0.85 (0.387–1.813)	0.683
Minimal diameter of radial artery (mm)	0.948 (0.422–2.172)	0.898
Categorical features		
Feature	OR	*p*-value
Sex (1—male, 0—female)	0.999 (0.485–2.032)	0.997
Recognized IHD	0.993 (0.448–2.085)	0.986
Diabetes	0.951 (0.453–2.113)	0.898
Hypertension	1.739 (0.725–3.87)	0.191
Hyperlipidemia	1.936 (0.942–3.968)	0.07
Smoking	1.517 (0.639–4.204)	0.378
History of CVA	>1000 (0–>1000)	0.985
History of MI	0.814 (0.362–2.019)	0.635
PCI performed previously	0.711 (0.314–1.772)	0.435
History of PCI	0.752 (0.342–1.788)	0.494
History of CABG	>1000 (0–>1000)	0.989
Diagnosis on admission: arteriosclerosis	1.744 (0.767–4.496)	0.211
Diagnosis on admission: unstable angina	3.576 (1.033–22.562)	0.088
Diagnosis on admission: NSTEMI	>1000 (0–>1000)	0.987
Diagnosis on admission: HD	1.237 (0.453–4.35)	0.705
Diagnosis on admission: other	1.229 (0.486–3.767)	0.687
Right radial artery	2.059 (0.448–7.032)	0.287
Heparin administered	0.55 (0.243–1.163)	0.131
Coronarography conducted	0 (0–>1000)	0.99
IVUS examination conducted	1.441 (0.263–26.852)	0.732
FFR measurement conducted	0.843 (0.217–5.567)	0.827
Vascular sheath 5F	6.121 (2.335–21.042)	0.001
Vascular sheath 6F	0.179 (0.052–0.47)	0.002
Catheter 6F	1.318 (0.63–2.914)	0.476
TIG catheter	0.736 (0.283–2.299)	0.559
Spasm occurrence	0.22 (0.094–0.538)	0.001
Spasmolytic administered	0.151 (0.052–0.452)	<0.001
Relanium administered	0.85 (0.139–16.311)	0.882
Isoptin administered	>1000 (0–>1000)	0.989
NTG administered	0.342 (0.071–2.457)	0.211
Conversion to transfemoral access	0.705 (0.109–13.73)	0.753
Pathological IMT	0.997 (0.396–2.302)	0.994
High origin of the radial artery	1.007 (0.268–6.565)	0.993
Atherosclerotic changes in radial artery	0.331 (0.155–0.726)	0.005
Presence of hematoma	>1000 (0–>1000)	0.987
False aneurysm	>1000 (0–>1000)	0.987

Abbreviations: SD—standard deviation; BMI—body mass index; eGFR—estimated glomerular filtration rate; EF—ejection fraction; CVA—cerebral vascular accident; PCI—percutaneous coronary intervention; CABG—coronary artery bypass graft surgery; MI—myocardial infarction; HD—heart defect; IVUS—intravascular ultrasound; FFR—fractional flow reserve; TIG—tiger; IMT—intima-media complex thickness.

## Data Availability

Data can be made available upon request.
